# Ethanol extract of *Ophiorrhiza pumila* suppresses liver cancer cell proliferation and migration

**DOI:** 10.1186/s13020-020-0291-4

**Published:** 2020-01-31

**Authors:** Hui Liu, Wanqin Liao, Lixia Fan, Zhaoguang Zheng, Dahai Liu, Qing-Wen Zhang, Anping Yang, Fang Liu

**Affiliations:** 1 Department of Basic Medicine and Biomedical Engineering, School of Stomatology and Medicine, Foshan University, Foshan, People’s Republic of China; 2 State Key Laboratory of Quality Research in Chinese Medicine, Institute of Chinese Medical Sciences, University of Macau, Macao Sar, People’s Republic of China

**Keywords:** *Ophiorrhiza pumila*, Liver cancer, Proliferation, Apoptosis, Migration

## Abstract

**Background:**

*Ophiorrhiza pumila,* belonging to the genus *Ophiorrhiza* (Rubiaceae), is distributed throughout tropical and subtropical Asia. In this study, we evaluated for the first time the anti-proliferation and anti-migration effects of ethanol extract of *O. pumila* (OPE) on HepG2 and SMMC-7721 cells, and explored the related mechanism.

**Methods:**

OPE was prepared by percolation with 95% ethanol and its main compounds were analyzed by HPLC-MS^2^. The anti-proliferation effect of OPE was evaluated by the CCK-8 assay and colony formation assay. Cell cycle distribution, apoptosis, and reactive oxygen species (ROS) level were detected by flow cytometry. Migration and invasion abilities were detected by Transwell migration/invasion assays. The expression of correlated proteins was determined using western blotting.

**Results:**

A total of 5 tentative compounds were identified from OPE, including pumiloside, deoxypumiloside, camptothecin, aknadinine, and β-stigmasterol. OPE displayed strong cytostatic effects on HepG2 and SMMC-7721 cells. OPE induced G2/M phase cell cycle arrest, increased apoptosis, and augmented ROS production in these cell lines. In addition, OPE possessed a significant inhibition on cell migration and invasion by reduction of MMP-9 and MMP-2 expression. Moreover, OPE significantly suppressed the phosphorylation of p65.

**Conclusions:**

Our data showed that OPE suppresses liver cancer cell proliferation and migration, which is possibly involved with the inhibition of the NF-κB pathway.

## Background

Liver cancer is one of the common malignant tumors in human and the second leading cause of cancer-related death around the world. Surgery and chemotherapy are customary therapeutic approaches for liver cancer [1]. Although surgical resection is able to remove lesions at early stage, high recurrence and metastasis usually occur after surgery. Chemotherapy is an important therapeutic strategy after surgery but its usage is limited due to drug resistance [2]. In recent years, immunotherapy has is supposed to be an attractive treatment investigated in liver maligancy. However, resistance to immune checkpoint blockades has been observed in most cancer patients [3, 4]. Therefore, it is still urgently needed to develop novel strategies for the management of liver cancer.

Nature products are important source of anti-cancer agents [5–8]. The genus *Ophiorrhiza,* a member of Rubiaceae family, is distributed throughout tropical and subtropical Asia. This genus encompasses approximately 150 species, some of them have been used as traditional medicines for the treatment of cough, myalgia, injuries, etc. [9–11]. Plants of this genus are rich source of camptothecin (CPT), a well-known anti-cancer drug [12, 13]. *Ophiorrhiza pumila,* belonging to the *Ophiorrhiza* genus, is an important herb cultivated in Guangdong, Fujian and Guangxi Provinces of China. It has been utilized in folk to treat fever, cold, cough. Previous phytochemical studies on *O. pumila* had resulted in the isolation of alkaloids, anthraquinones and glucosides. Some reports of *O. pumila* focused on measurement and improvement of the content of CPT in this plant [10, 11, 14–16]. However, the full scope of the anti-cancer effects of *O. pumila*’s compounds remains largely unknown.

In this study, we sought to analyze the main compounds of ethanol extract of *O. pumila* (OPE) by HPLC-MS^2^ and investigate the related anti-tumor activity in liver cancer cells, which may provide experimental evidence for extensive mechanism exploring and contribute to the utilizing of *O. pumila*.

## Methods

### Reagents and materials

Human Bax, Bcl-2 and cleaved caspase-3 antibodies were from Cell Signaling Technology (USA). Cyclin D1, Cyclin A, Cyclin B1, CDK1, phospho-p65, phospho-ERK, phospho-AKT, MMP-2, MMP-7, and MMP-9 antibodies were purchased from Proteintech (USA). GAPDH antibody and HRP-conjugated secondary antibodies were purchased from BIOSS (Beijing, China). *Ophiorrhiza pumila* was purchased from Foshan Renhui Pharmaceutical Technology Co. (Foshan City, Guangdong Province, China).

### Preparation of OPE and HPLC-MS^2^ analysis

The dried whole plant of *O. pumila* (100 g) were crushed, and extracted by 95% (v/v) ethanol for three times (3 × 1.5 L) at room temperature. The combined extract solution was concentrated under vacuum and produced a total of 5.2 g ethanol extract (OPE). OPE was stored at − 20 °C before use.

HPLC-MS^2^ analysis was performed on an Angilent-1260 system coupled with a Bruck amaZon SL mass spectrometry. Chromatographic separation was performed on a reverse phase YMC-pack ODS-A-HG column (4.6 × 150 mm, 5.0 μm); Mobile phase composed of water and acetonitrile. The program of gradient elution was 15% acetonitrile at 0–15 min, 15–50% acetonitrile at 15–30 min, 50–95% acetonitrile at 30–40 min and 95% B at 40–55 min. The flow rate and the injection volume were 1 mL/min and 10 μL, respectively. The detection wavelengths were set up at 210 nm. The positive ion modes were used for the mass detection. The source parameters were set as follows: ion spray voltage, 4500 V; the flow rate of drying gas, 8 L/min; the temperature of drying gas, 220 °C; the spectra range, 100–1500 *m/z*.

### Cell culture

HepG2 and BRL3A cells were obtained from the American type culture collection (ATCC). SMMC-7721 cells were purchased from the Shanghai Cell Bank of the Chinese Academy of Sciences (China) (Additional file 1: Figure S1). Cells were maintained in DMEM medium containing 10% fetal bovine serum (FBS) and 1% penicillin/streptomycin and in a humidified 5% CO_2_ atmophere at 37 °C.

### Cell viability assay

HepG2, SMMC-7721, and BRL3A cells (4–9 × 10^3^ cells per well) were seeded into a 96-well plate in 100 μL culture medium and treated with OPE (0, 1.56, 3.125, 6.25, and 12.5 μg/mL) for 24 h, 48 h, and 72 h, respectively. Then, the viability was determined by CCK-8 (Cell Counting Kit-8) (Dojindo, Japan). Briefly, at the end of the incubation time, 10 μL of CCK-8 solution (10 μL/well) was added and maintained at 37 °C for 1–4 h. Absorbance was measured at 450 nm.

### Colony formation assay

Liver cancer cells were added into 6-well plates and treated with OPE (0, 0.3, 0.6, 0.9, 1.2, and 1.5 µg/mL) for 48 h. Then 500–800 cells were seeded in 6-well plated and culture up to 12–14 days. After fixing with cold methanol, cells were stained with 0.5% crystal violet solution. Colonies were photographed under a microscope, and the number of colonies was counted.

### Apoptosis analysis and Hoechst 33258 staining

The apoptosis inducing effect of OPE was addressed by flow cytometry. In brief, HepG2 and SMMC-7721 cells (2–3 × 10^5^ cells per well) were plated in 6-well plates and treated with OPE (0, 3.125, and 6.25 μg/mL) for 48 h at 37 °C. Thereafter, cells were washed with PBS, and stained with Annexin-V-FITC (5 μL) and PI (2 μL) for 10 min. Finally, stained cells were examined by flow cytometry. For Hoechst 33258 staining, HepG2 and SMMC-7721 cells treated with OPE for 48 h. After fixing with 4% PFA, cells were incubated with Hoechst 33258 solution (5 μg/mL) for 15 min. The morphology of nuclei was detected under a fluorescence microscope (Zeiss, Germany).

### Determination of intracellular ROS

The production of ROS was determined by DCFH-DA staining. After treatment with OPE (0, 3.125, and 6.25 μg/mL) for 48 h, cells collected and incubated in a 5 μg/ml DCFH-DA solution at 37 °C for 30 min in the dark. Then cells were washed twice with PBS, and the stained cells were analyzed by flow cytometry.

### Cell cycle analysis

The potential of OPE to arrest the cell cycle of HepG2 and SMMC-7721 cells was evaluated by flow cytometry. Cells (2 × 10^5^ cells per well) were seeded in 6-well plates and incubated for 12 h, followed by incubation with 0, 3.125, and 6.25 μg/mL of OPE for 48 h. Then cells were harvested, and stained with PI using a Cell cycle staining Kit (Multi Sciences, China). The fluorescent emission was measured by flow cytometry.

### The migration and invasion assay

Cell migration assay was performed using Transwell apparatus (Corning). After treatment with OPE (0, 0.78, 1.56, 3.125, and 6.25 μg/mL) for 48 h, cells (3–5 × 10^4^) in 100 μL medium without FBS was added to the upper chamber while 600 μL medium containing 10% FBS was added to the lower chamber. After incubation for 24 h, cells on the upper surface of the filter were removed. Filters were fixed with methanol and stained with crystal violet (0.5%). Then chose three fields and counted under a light microscope. For invasion assay, before cell plating, the filters were treated with using Matrigel (BD Biosciences, Germany) and the following procedures were performed as the migration assay.

### Western blotting

Cells were treated with OPE (0, 3.125, and 6.25 μg/mL) for 48 h. Then cells were washed twice with cold PBS and subsequently lysed in RIPA buffer. Protein concentration was accessed by using BCA protein Assay Kit (Millipore, USA). Proteins were separated by 10–12% SDS-PAGE, and transferred onto polyvinylidene fluoride (PVDF) membranes. After blocking, membranes were incubated with antibodies against Bax, Bcl-2, cleaved caspase-3, MMP-2, MMP-7, MMP-9, Cyclin D1, Cyclin A, Cyclin B1, CDK1, p-p65, p-ERK, p-AKT, and GAPDH at 4 °C overnight. After washing with TBST three times, membranes were incubated with HRP-conjugated secondary antibodies at room temperature for 1 h. The protein bands were detected by using an ECL kit (Millipore). Protein was quantified by Image J software.

### Statistical analysis

All experiments were performed in triplicated. The results were expressed as mean ± SD. The one-way analysis of variance (ANOVA) test was used to compare the data. A *p* < 0.05 was considered statistically significant.

## Results

### Analysis of the main components in OPE by HPLC-MS^2^

**Fig. 1:**
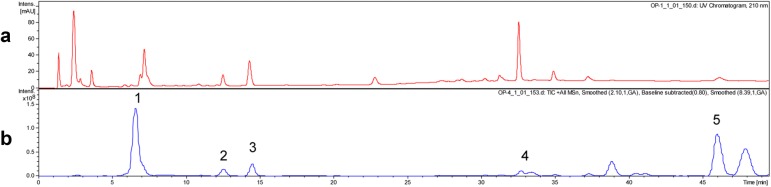
HPLC-MS^2^ analysis of the OPE. **a** UV chromatogram (210 nm). **b** Total ion chromatogram (positive ion mode)

To defined the components of OPE, HPLC-MS^2^ analysis was performed. The UV chromatography and total ion current chromatogram in positive ESI mode are shown in Fig. 1. A total 5 tentative compounds, including pumiloside, deoxypumiloside, camptothecin, aknadinine, and β-stigmasterol were identified by analyzing the molecular weight and fragmentation behavior. Their relative peak area detected by HPLC at 210 nm were also obtained by the integral method (Table 1) [9, 16, 17].

**Table 1:** Compounds identified from the chromatogram of OPE by HPLC-MS^2^

Peak	Retention time (min)	MS (*m/z*)	MS^2^ (*m/z*)	Tentative compounds	Relative peak area (%)
1	7.2	513.26	351.16	Pumiloside	4.37
2	12.6	497.25	335.19	Deoxypumiloside	3.39
3	14.5	349.11	337.19	Camptothecin	8.48
4	32.5	383.26	327.15	Aknadinine	16.0
5	46.1	413.31	301.11	β-Stigmasterol	2.42

### The effect of OPE on the proliferation of liver cancer cells

**Fig. 2:**
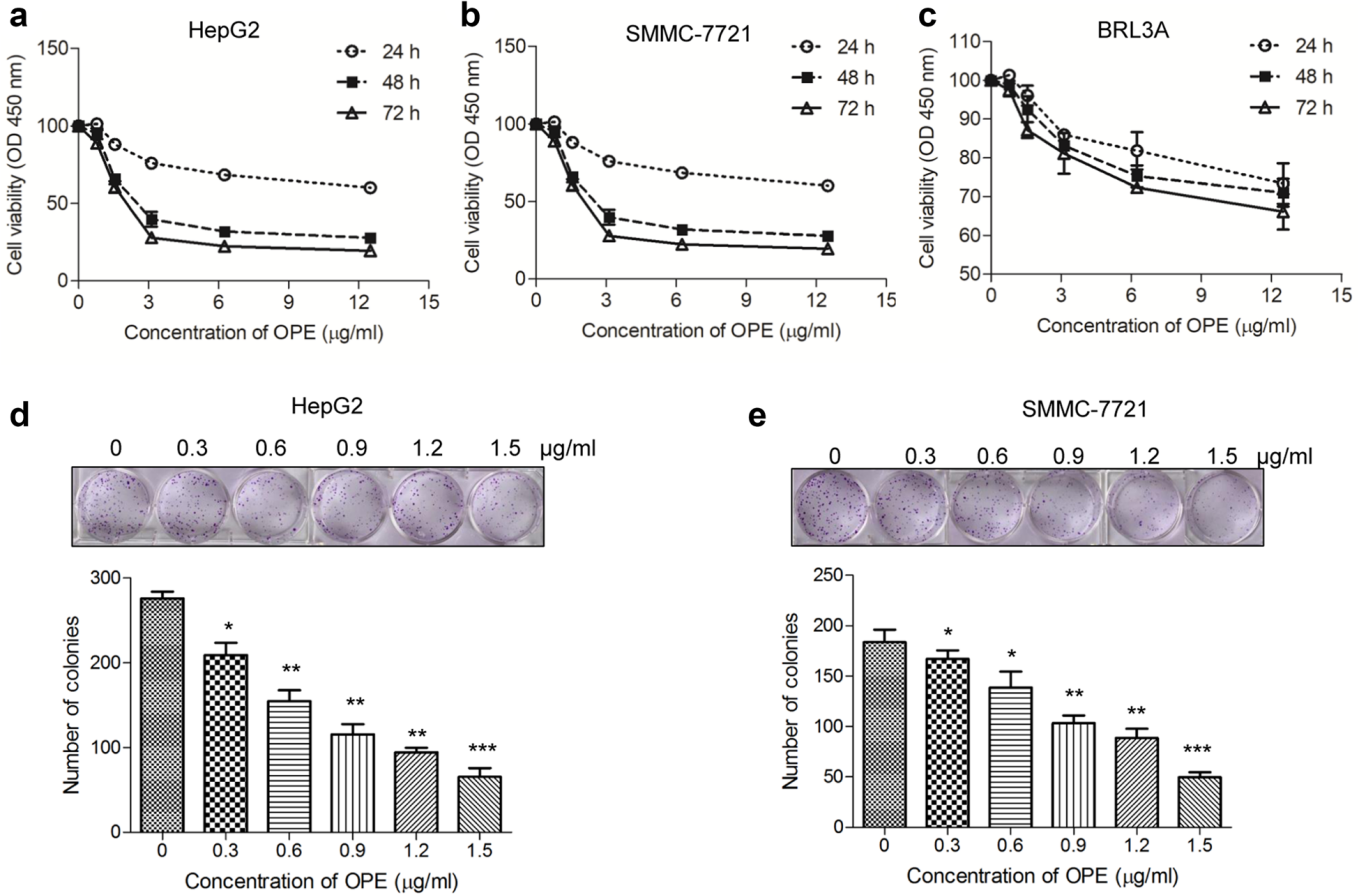
The anti-proliferative effect of OPE on cells. **a–c** HepG2, SMMC-7721 and BRL3A cells were exposed to designated concentrations (0–12.5 µg/mL) of OPE for 24, 48, and 72 h. Cell viability was determined by CCK-8 assay. **d**, **e** The effect of OPE (0–1.5 µg/mL) on colony formation in liver cancer cells. The statistic results of colony formation assays presented as surviving colonies. Data are presented as mean ± SD of at least three independent experiments. (*p < 0.05; **p < 0.01;***p < 0.001, compared to untreated control)

To evaluate the anti-proliferative effect of OPE, normal cells BRL3A, human liver cancer cells HepG2 and SMMC-7721 were treated with various concentrations of OPE (0, 1.56, 3.125, 6.25, and 12.5 μg/mL) for 24, 48, and 72 h, respectively, and cell proliferation was determined by CCK-8 assay. We found that OPE exhibited a significant inhibitory effect on the proliferation of HepG2 and SMMC-7721 cells in a time and dose-dependent manner (Fig. 2a, b). The IC_50_ values at 24, 48, and 72 h time points were 21.7, 2.1, and 1.4 µg/mL for HepG2 cell; 13.6, 1.6, and 1.1 µg/mL for SMMC-7721 cells, respectively. In addition, the normal rat liver cell line BRL3A was used to assess the cytotoxic effect of OPE. The result showed that OPE exhibited lower cytotoxicity in BRL3A cells in comparison with HepG2 and SMMC-7721 cells, indicating that OPE selectively inhibits the growth of liver cancer cells (Fig. 2c). To further access the long-term effect of OPE on liver cancer cell survival, the colony formation assay was conducted. Diminishing cells were observed as the concentration of OPE increased (Fig. 2d, e). Together, these data indicated that OPE displays strong cytostatic effect on HepG2 and SMMC-7721 cells.

### OPE induces G2/M arrest in liver cancer cells

**Fig. 3:**
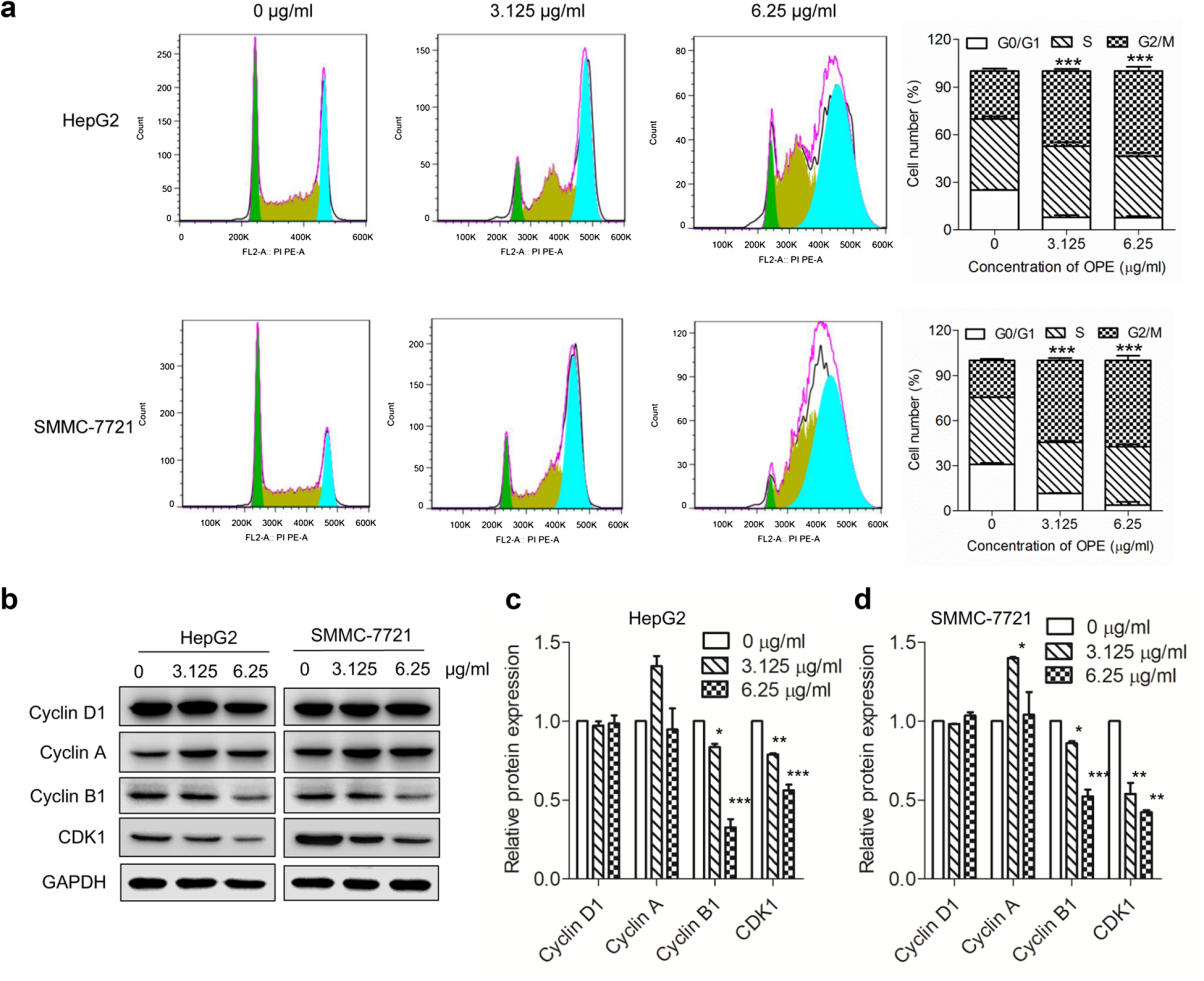
OPE induces G2/M phase cell cycle arrest in liver cancer cells. **a** HepG2 and SMMC-7721 cells were treated with designated concentrations of OPE (0, 3.125, and 6.25 μg/mL) for 48 h and the cell cycle distribution was determined by a flow cytometric analysis. **b** Western blot analysis of protein expression levels of Cyclin D1, Cyclin A, Cyclin B1, and CDK1 in HepG2 and SMMC-7721 cells treated with OPE (0, 3.125, and 6.25 μg/mL) for 48 h. GAPDH was served as a loading control. **c**, **d** The relative protein expression of Cyclin D1, Cyclin A, Cyclin B1, and CDK1. Data are presented as mean ± SD of at least three independent experiments. (*p < 0.05; **p < 0.01;***p < 0.001, compared to untreated control)

To elucidate the mechanism by which OPE suppresses cell proliferation, the effect of OPE on cell cycle progression was investigated. As shown in Fig. 3a, OPE induced increased population of cells at the G2/M phase, which was in a dose-dependent manner. At the concentration of 6.25 µg/mL, the G2/M enrichment was enhanced from 30.2% (control) to 53.7% (*p* < 0.001) in HepG2 cells. Similarly, an increase in G2/M accumulation from 24.6% (control) to 57.4% (*p* < 0.001) was observed in SMMC-7721 cells treated with 6.25 µg/mL OPE. The arrest of the liver cancer cells at G2/M phase was further confirmed by western blotting with cell cycle-related proteins. It was found the expression levels of G2/M regulators, Cyclin B1 and CDK1, were markedly decreased after treatment with OPE (Fig. 3b–d). These data revealed that OPE inhibits the growth of liver cancer cells by inducing cell cycle arrest at G2/M phase.

### OPE induces apoptosis in liver cancer cells

**Fig. 4:**
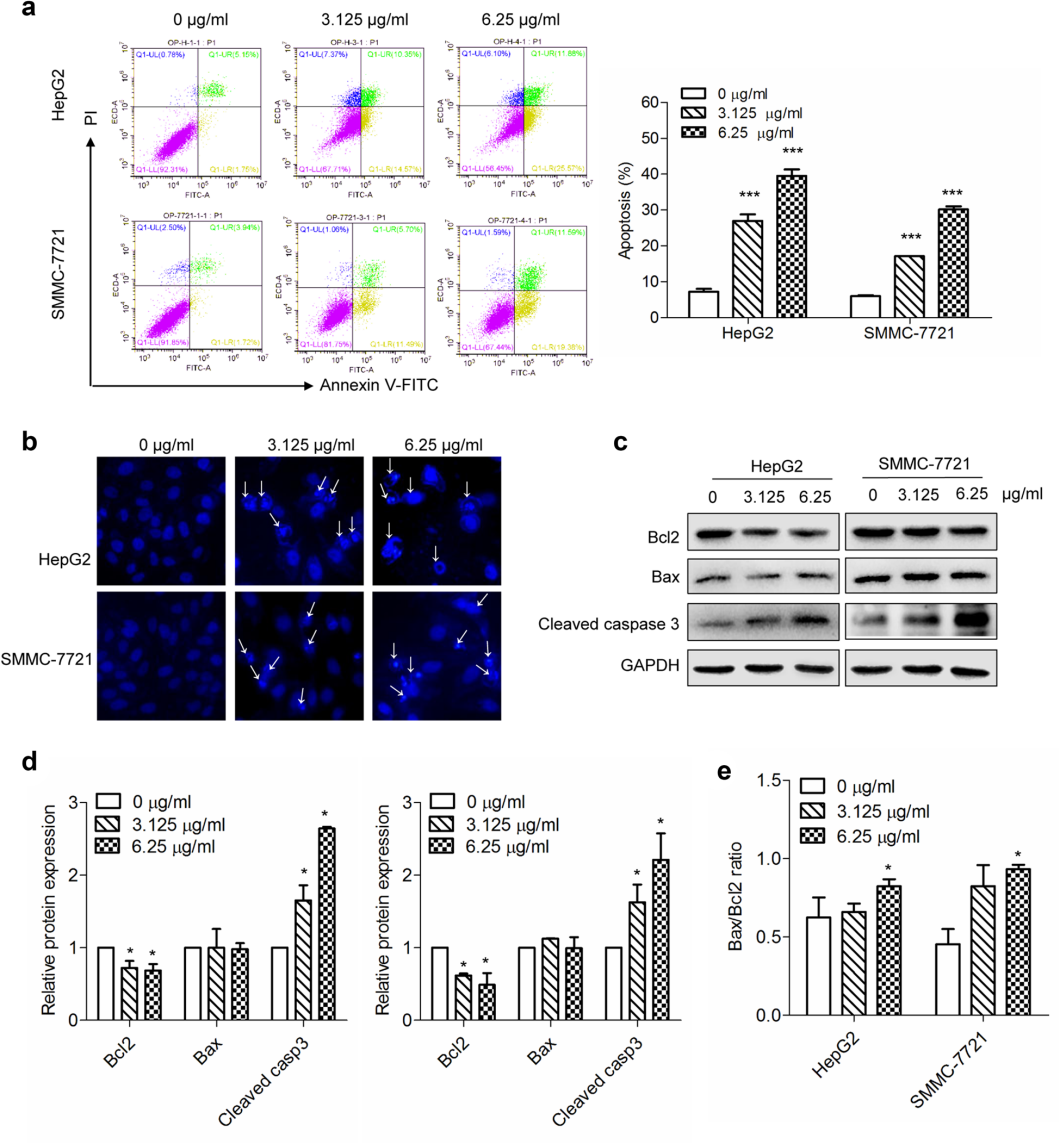
OPE induces apoptosis in liver cancer cells. **a** HepG2 and SMMC-7721 cells were incubated with OPE at different doses for 48 h. The apoptosis rate was treated statistically. **b** Morphological features of nuclei of HepG2 and SMMC-7721 cells treated with various concentrations of OPE for 48 h (20 ×). **c** Western blot analyses of HepG2 and SMMC-7721 cells treated with OPE (0, 3.125, and 6.25 μg/mL) for 48 h to evaluate protein expression of Bax, Bcl-2, cleaved caspase-3. GAPDH was served as a loading control. **d** The relative protein expression of Bcl-2, Bax and cleaved caspase-3. **e** The percentage of Bax/Bcl-2 ratio. Data are presented as mean ± SD of at least three independent experiments. (*p < 0.05; ***p < 0.001, compared to untreated control)

To verify whether OPE induces apoptosis in HepG2 and SMMC-7721 cells, flow cytometry analysis were applied. The apoptosis rate was markedly increased from 7.2%, to 27.0% (*p* < 0.001) and 39.5% (*p* < 0.001) for HepG2 cells and from 6.0% to 17.1% (*p* < 0.001) and 30.2% (*p* < 0.001) for SMMC-7721 cells after 48 h OPE-treatment (Fig. 4a). Accordingly, increased fluorescence intensities of nuclei and nuclear condensation were observed in HepG2 and SMMC-7721 cells upon OPE treatment in Hoechst 33258 staining (Fig. 4b). To further access the preliminary pathways of OPE-induced apoptosis, the levels of Bcl-2, Bax, as well as cleaved caspase-3 were determined by western blotting. The results showed that OPE treatment resulted in a significantly decrease in the expression of anti-apoptotic protein Bcl-2 and a remarkable increase in the expression of pro-apoptotic Bax and cleaved caspase-3 (Fig. 4c, d). Moreover, the ratio of Bax/Bcl-2 was also significantly elevated (Fig. 4e). Together, these data proved that OPE strongly induces apoptosis in HepG2 and SMMC-7721 cells.

### The effect of OPE on ROS of liver cancer cells

**Fig. 5:**
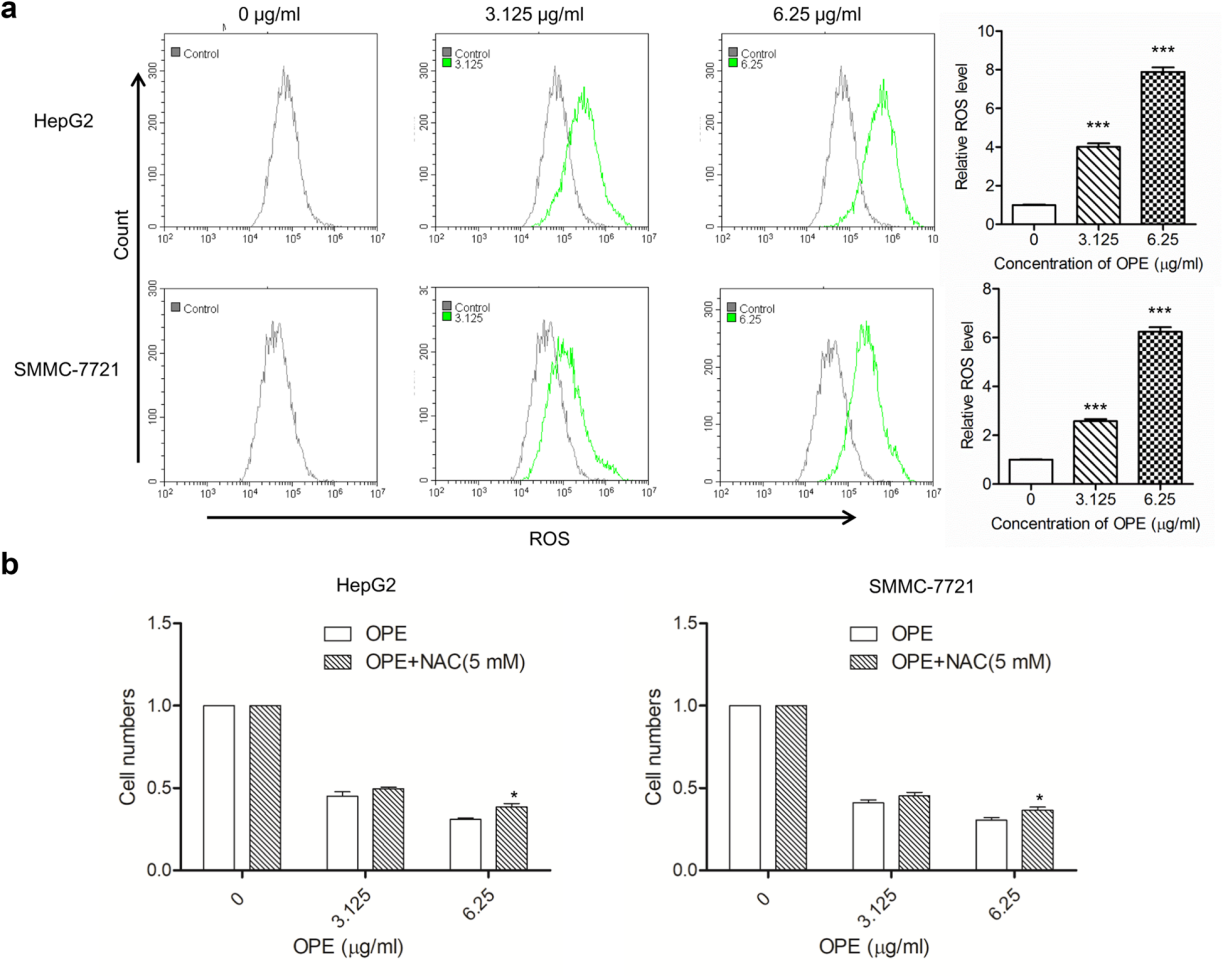
ROS involvement in OPE-induced liver cancer cell apoptosis. **a** HepG2 and SMMC-7721 cells were treated with OPE at indicated doses (0, 3.125, and 6.25 μg/mL) for 48 h, followed by analysis of ROS by flow cytometry. **b** Cells were treated with OPE plus NAC (5 mM) for 48 h, and cell viability was determined by using CCK-8 assay. Data are presented as mean ± SD of at least three independent experiments. (*p < 0.05; ***p < 0.001, compared to untreated control)

Increased ROS production is considered to be an important factor controlling cell survival and apoptosis [18–20]. The effect of OPE on ROS generation was examined using DCFH-DA staining. As indicated in Fig. 5a, OPE significantly increased ROS level in a dose-dependent manner. OPE significantly increased ROS level to two- to fourfold (*p* < 0.001) at concentration of 3.125 μg/mL, and six- to eightfold (*p* < 0.001) at concentration of 6.25 μg/mL. To confirm the role of ROS involved in OPE-mediated cytotoxicity, cell viabilities were measured in cells treated with OPE at concentrations of 3.125 and 6.25 μg/mL plus NAC (5 mM). As shown in Fig. 5b, OPE-induced cell death was partially reversed by NAC at the concentration of 6.25 μg/mL. Thus, these results revealed that OPE-induced inhibition in the viability of HepG2 and SMMC-7721 cells is associated with changes of intracellular ROS production.

### Effects of OPE on the migration and invasion of liver cancer cells

**Fig. 6:**
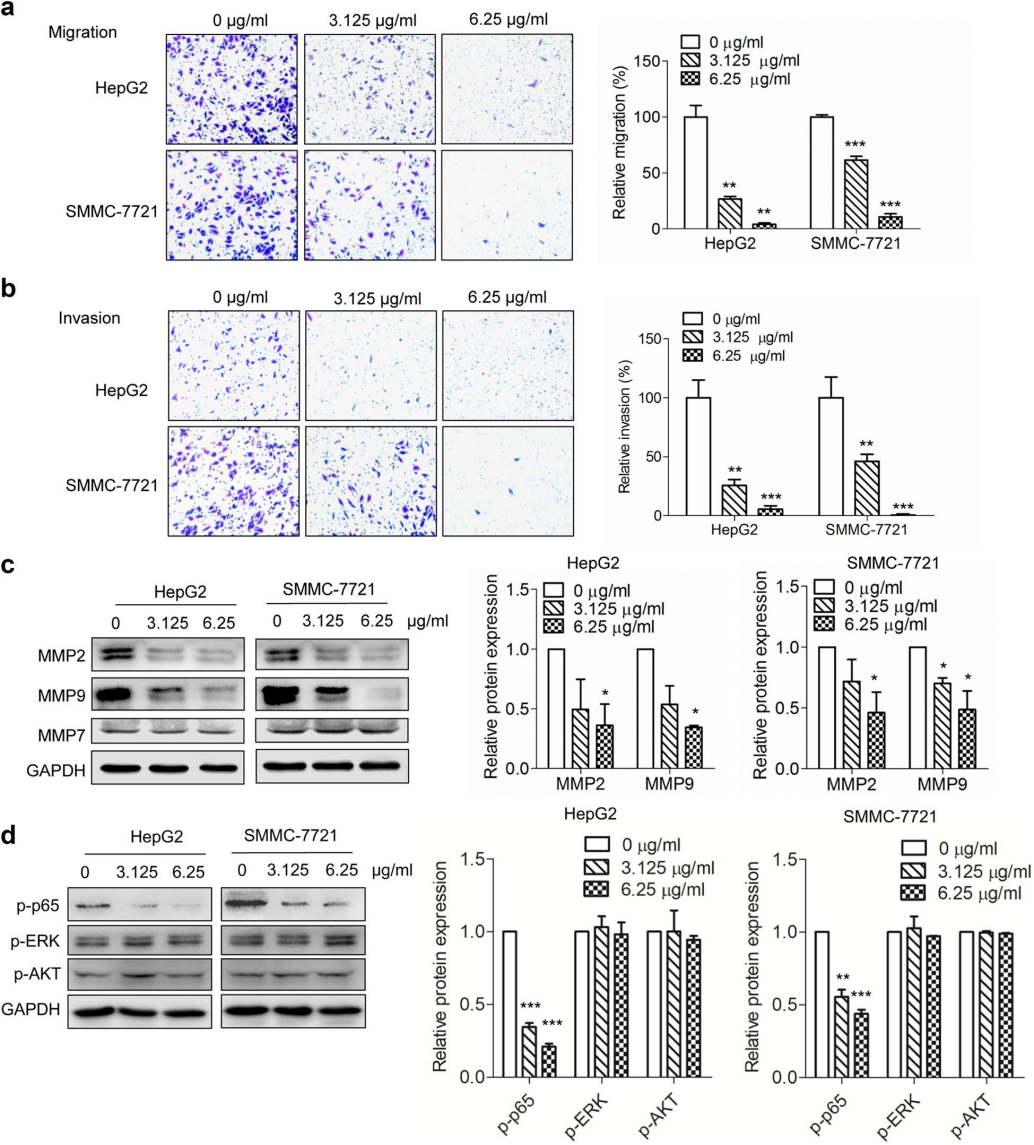
Inhibitory effects of OPE on cell migration and invasion of liver cancer cells. **a**, **b** HepG2 and SMMC-7721 cells were treated with different concentrations of OPE (0, 3.125, and 6.25 μg/mL) for 48 h, migrated and invasive cells were photographed and quantified (20 ×). **c** Western blot was carried out to detect the expression of migration-associated proteins (MMP-2, MMP-9 and MMP-7). **d** Western blot was carried out to detect the phosphorylation levels of p65, ERK, and AKT. GAPDH served as loading control. Data are presented as mean ± SD of at least three independent experiments. (*p < 0.05; **p < 0.01; ***p < 0.001, compared to untreated control)

A leading cause of death in patients with cancer is tumor metastasis [21]. Cell migration and adhesion are two critical steps implicated in the progression of cancer metastasis. To investigate the potential effects of OPE on liver cancer metastasis, transwell migration and invasion assay were performed in HepG2 and SMMC-7721 cells. The migration of the two cell lines was concentration-dependently inhibited by OPE exposure (Fig. 6a and Additional file 2: Figure S2a). The invasive capabilities of HepG2 and SMMC-7721 cells were also significantly decreased after OPE treatment (Fig. 6b and Additional file 2: Figure S2b).

Matrix metalloproteinases (MMPs) are integral in basement membrane degradation during the progress of tumor cell migration and invasion [22]. We performed western blotting to preliminary study the effect of OPE on the protein expression of several key proteins in liver cancer cells. As indicated in Fig. 6c, OPE treatment induced large concentration dependent reductions of MMP-2 and MMP-9 protein expression, but had no significant effect on MMP-7 expression. Taken together, these results suggested that OPE possesses significant inhibitory effects on the migration and invasion of HepG2 and SMMC-7721 cells.

NF-κB, AKT and ERK pathways have been proved play an important role in cancer cell proliferation and migration [23–25]. Thus, we investigated whether OPE has an effect on these pathways. The results from western blot analysis showed that OPE significantly decreased the phosphorylation of p65, but had no significant effect on the levels of phospho-ERK and phospho-AKT (Fig. 6d), indicating that OPE may inhibit cancer cell proliferation and motility by inactivating NF-κB signaling.

## Discussion

For most traditional Chinese medicines, the active ingredient may be a mixture of certain ingredients [26]. *O. pumila,* is belonging to the *Ophiorrhiza* plants which are important components of traditional medicines for the treatment of various disease, such as cough, myalgia, and injury, indicating a critical medical value of these plants [9, 10, 14]. Previous phytochemical studies on *O. pumila* indicated that it is composed of anthraquinones, glucosides and chlorogenic acid, in addition to alkaloids [12, 15, 27, 28]. In this study, we first identified the chemical constituents of OPE using HPLC-MS^2^ method. In combination with the previous articles and MS fragmentation behavior, chemical analysis of OPE indicated that the main components were pumiloside, deoxypumiloside, camptothecin, aknadinine, and β-stigmasterol.

Some studies reported the development and biosynthesis of CPT in *O. pumila* [16], but the total effects of *O. pumila*’s compounds (OPE) remains largely unknown. In the present study, the anti-proliferation effect of OPE was explored for the first time on liver cancer cells in vitro. Two human liver cancer cell lines (HepG2 and SMMC-7721) were used. OPE showed significantly cytotoxicity against the liver cancer cells in a time and concentration-dependent manner. Most notably, OPE displayed a rather low IC_50_ value of 2.06 μg/mL and 1.58 μg/mL against HepG2 and SMMC-7721 cell lines, respectively. It is known that cell cycle arrest is an important cause of inhibition of cancer cell proliferation. For example, β-cryptoxanthin has been reported to inhibit cell proliferation by inducing G0/G1 arrest in gastric cancer [29] and simvastatin induces G0/G1 arrest in HepG2 and Hep3B cells [30]. In our test, OPE caused the arrest of HepG2 and SMMC-7721 cells at G2/M cell cycle phase.

Accumulated evidence supports that anticancer drugs exert their cytotoxic effect mainly by inducing cancer cell apoptosis. For instance, the ethanol extract of *Moringa oleifera* leaf initiated apoptosis by down-regulation of Bcl-2 and Bcl-xL and up-regulation of Bax and caspase-3 [31]. Coptisine induces apoptosis of human colon cancer cells via mitochondrial-associated apoptotic pathway mediated by PI3K/Akt [32]. In the present study, OPE was found to enhance apoptosis in HepG2 and SMMC-7721 cells. The extrinsic and intrinsic pathways are the two main pathways in apoptosis. The extrinsic pathway is activated by the binding of death ligands (e.g., TNF-α, and CD95L/FasL). In the intrinsic apoptosis, pro-apoptotic proteins (e.g., Bax, Bad, and Bid) and anti-apoptotic proteins (e.g., Bcl-2, and Bcl-XL) have been shown to play important roles [33–35]. Our data showed that OPE caused substantial increase in the expression of cleaved caspase-3 and Bax and decrease in the expression of Bcl-2 in both HepG2 and SMMC-7721 cells, which may provide some primary mechanisms of apoptosis induced by OPE.

Extensive studies have indicated that ROS play a crucial role in the proliferation of cancer cells. Abnormal accumulation of ROS may effects cell viability via damage some macromolecules, including peroxidation of membrane lipids, protein denaturation and DNA damage [20]. In this study, the obvious increase in the intracellular ROS level indicated that OPE induced suppression of HepG2 and SMMC-7721 cell viability partially by inducing ROS generation.

The migratory and invasive ability are related to the metastatic potential of cancer cells, which contribute to cancer progression and poor outcomes of patients [36, 37]. In this study, we observed that OPE dose-dependently inhibited the migration and invasion of the HepG2 and SMMC-7721 cells, which concomitant with the decrease in the expression of MMP-2 and MMP-9. Additionally, OPE significantly repressed the phosphorylation of p65, indicating the OPE may exert its cytotoxic effect by downregulation of NF-κB signaling.

## Conclusions

Our study demonstrated that OPE mediated cell growth suppression by inducing cell cycle arrest and enhancing apoptosis and ROS accumulation. In addition, OPE exhibited anti-migration and anti-invasion effects on liver cancer cells, which might be related to decreased expression of MMP-2 and MMP-9. Moreover, OPE had a suppressive effect to NF-κB signaling, which may contributed to the cytotoxic activity of OPE.

## Supplementary information

**Additional file 1: Figure S1.** STR profiling of SMMC-7721 and HepG2 cells.

**Additional file 2: Figure S2.** Inhibitory effects of OPE on cell migration and invasion of liver cancer cells. **a**, **b** HepG2 and SMMC-7721 cells were treated with different concentrations of OPE (0, 0.78, and 1.56 μg/mL) for 48 h, and the migrated and invasive cells were photographed and quantified.

## Data Availability

All data used to support the findings of this study are available from the corresponding author upon request.

## Abbreviations

OPE: ethanol extract of *O. pumila*
HPLC-MS^2^: high performance liquid chromatography with mass spectrometry
CCK-8: cell counting kit-8
ROS: reactive oxygen species
CPT: camptothecin
MMP-2: matrix metalloproteinase-2
MMP-7: matrix metalloproteinase-7
MMP-9: matrix metalloproteinase-9
GAPDH: glyceraldehyde-3-phosphate dehydrogenase
DCFH-DA: 2,7-dichlorodi-hydrofluor- escein diacetate
Bcl-2: B cell lymphoma/lewkmia-2
Bcl-xL: recombinant human B cell leukemia/lymphoma 2 XL
TNF-α: tumor necrosis factor-α
