# Supplementary figures and images for: Ethanol extract of Ophiorrhiza pumila suppresses liver cancer cell proliferation and migration
Source: Chin Med. 2020 Jan 31;15:11. doi: 10.1186/s13020-020-0291-4 (PMC6995237; doi:10.1186/s13020-020-0291-4)

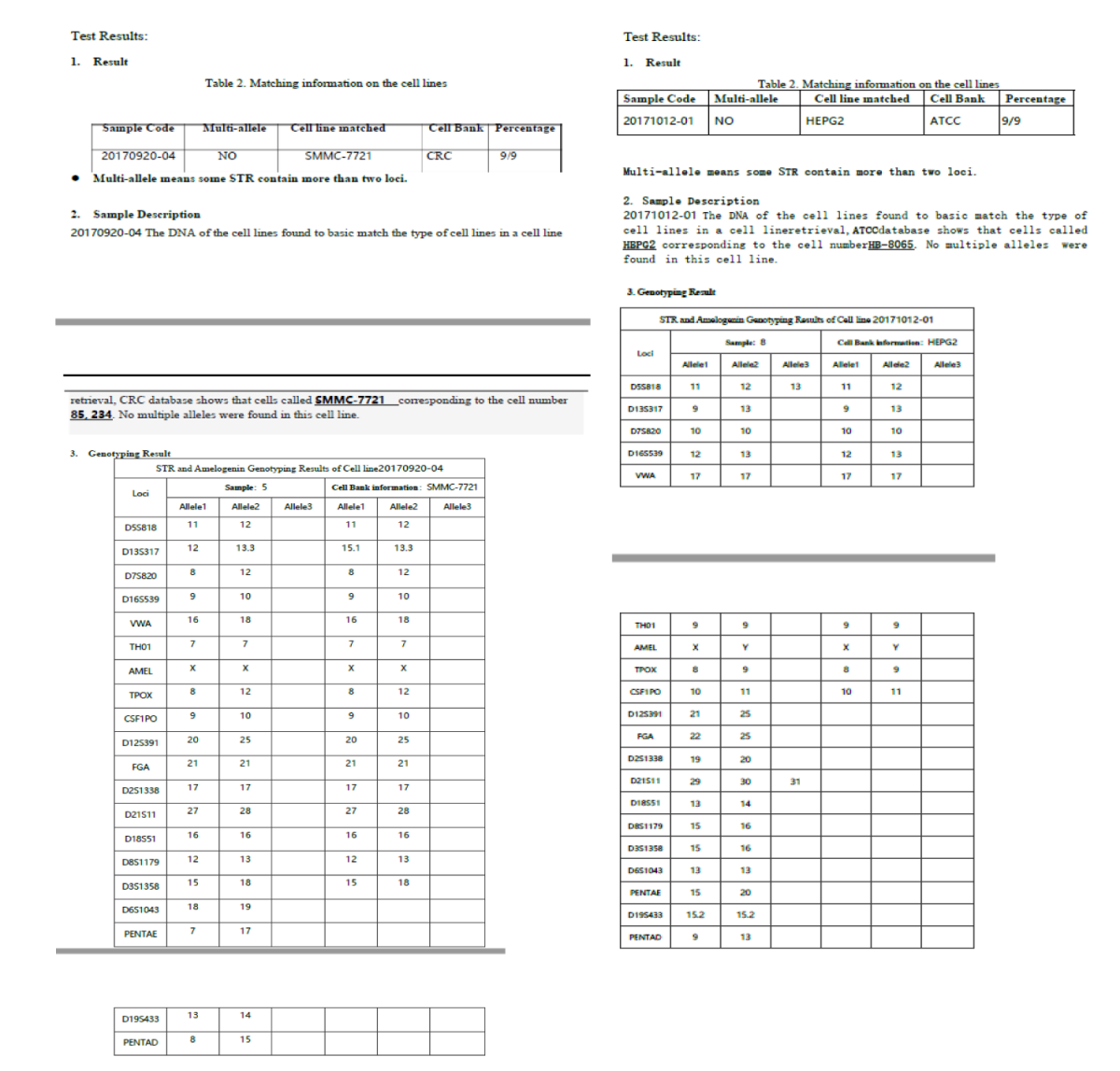


Figure S1 STR profiling of SMMC-7721 and HepG2 cells.

Supplement: Supplementary file 1 — Additional file 1: Figure S1. STR profiling of SMMC-7721 and HepG2 cells. [file 13020_2020_291_MOESM1_ESM.docx]
