# Supplementary material for: Ethanol extract of Ophiorrhiza pumila suppresses liver cancer cell proliferation and migration
Source: Chin Med. 2020 Jan 31;15:11. doi: 10.1186/s13020-020-0291-4 (PMC6995237; doi:10.1186/s13020-020-0291-4)
Supplement: Supplementary file 2 — Additional file 2: Figure S2. Inhibitory effects of OPE on cell migration and invasion of liver cancer cells. a, b HepG2 and SMMC-7721 cells were treated with different concentrations of OPE (0, 0.78, and 1.56 μg/mL) for 48 h, and the migrated and invasive cells were photographed and quantified. [file 13020_2020_291_MOESM2_ESM.docx]

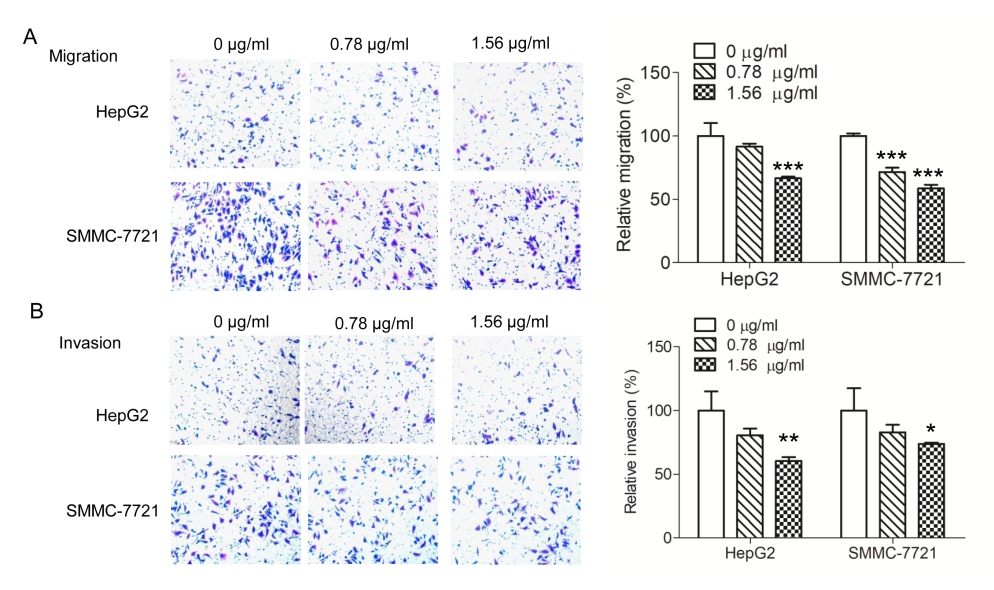


Figure S2 Inhibitory effects of OPE on cell migration and invasion of liver cancer cells. (A, B) HepG2 and SMMC-7721 cells were treated with different concentrations of OPE (0, 0.78, and 1.56 μg/mL) for 48 h, and the migrated and invasive cells were photographed and quantified.
